# Mundgesundheit im Pflegeheim als interprofessionelle Aufgabe: Ergebnisse einer qualitativen Befragung von Pflegefachpersonen und Zahnärzt*innen zu ihrer aktuellen und zukünftigen Zusammenarbeit

**DOI:** 10.1007/s00391-022-02132-5

**Published:** 2022-11-07

**Authors:** Marie Hamacher, Cornelia Weiß, Kerstin Hämel

**Affiliations:** 1 AG 6 Versorgungsforschung & Pflegewissenschaft, Fakultät für Gesundheitswissenschaften, Universität Bielefeld, Universitätsstr. 25, 33615 Bielefeld, Deutschland; 2 Stiftungsprofessur Rehabilitationswissenschaften | Rehabilitative Versorgungsforschung, Fakultät für Gesundheitswissenschaften, Universität Bielefeld, Bielefeld, Deutschland; 3 Institut für Rehabilitationsmedizin, Profilzentrum Gesundheitswissenschaften, Medizinische Fakultät der Martin-Luther-Universität Halle-Wittenberg, Halle (Saale), Deutschland

**Keywords:** Teambasiert, Langzeitversorgung, Qualitative Forschung, Zahnmedizinische Versorgung älterer Menschen, Erweiterte Pflegerolle, Team based, Long-term care, Qualitative research, Dental care for aged, Advanced nursing role

## Abstract

**Hintergrund:**

Menschen mit Pflegebedarf haben im Vergleich zu Gleichaltrigen ohne Pflegebedarf eine signifikant schlechtere Mundgesundheit. International wird diesen Herausforderungen verstärkt durch interprofessionelle Zusammenarbeit und erweiterte Rollen von Pflegefachpersonen begegnet. Dieser Beitrag untersucht die Sichtweisen von Zahnärzt*innen und Pflegefachpersonen in Deutschland auf ihre aktuelle und künftige Zusammenarbeit in Pflegeheimen.

**Methode:**

Es wurden 8 Experteninterviews mit jeweils 4 praktizierenden Zahnärzt*innen und Pflegefachpersonen aus der Region Westfalen-Lippe via Zoom oder telefonisch durchgeführt. Das vollständig transkribierte Interviewmaterial wurde mittels thematischem Kodieren fallbezogen und fallübergreifend ausgewertet.

**Ergebnisse:**

Für eine Förderung der Mundgesundheit von Heimbewohner*innen ist aus Sicht der Befragten die Zusammenarbeit von Zahnärzt*innen und Pflegefachpersonen unabdingbar. Sie schildern Zeit- und Kompetenzmangel in der Mund- und Zahnversorgung von Heimbewohner*innen, denen mit neuen Verantwortlichkeitsrollen für speziell qualifizierte Pflegefachpersonen in Kooperation mit den Zahnärzt*innen begegnet werden sollte. Zugleich sprechen sie sich für eine stärkere Verankerung und Einbettung (zahn)medizinischer Versorgung in den Abläufen in Pflegeheimen aus.

**Schlussfolgerung:**

Neue Kooperationsformen zwischen Zahnärzt*innen und Pflegefachpersonen in Pflegeheimen sollten in Deutschland erprobt und weiter ausgebaut werden.

**Zusatzmaterial online:**

Zusätzliche Informationen sind in der Online-Version dieses Artikels (10.1007/s00391-022-02132-5) enthalten.

## Abstract (en)

**Background:**

People with long-term care needs have significantly poorer oral health than their peers without care needs. Internationally, this is increasingly being addressed through interprofessional collaboration and expanded roles of nurses. This article investigates the perspectives of dentists and nurses in Germany on the current status and future of their collaboration in nursing homes.

**Methods:**

A total of eight expert interviews were conducted with four practising dentists and nurses from the Westphalia-Lippe region via Zoom or by telephone. The fully transcribed interview material was analyzed by thematic coding on a case-specific and cross-case basis.

**Results:**

From the perspective of the interviewees, collaboration of dentists and nurses is indispensable for promoting the oral health of nursing home residents. They describe a lack of time and competence in the oral and dental care of home residents, which should be countered with new roles of responsibility for specially qualified nurses in cooperation with dentists. At the same time, they advocate a stronger anchoring and embedding of (dental) medical care in the routines of nursing homes.

**Conclusion:**

New forms of cooperation between dentists and nurses in nursing homes should be piloted and further developed in Germany.

## Hintergrund

Die weltweit hohe Prävalenz oraler Erkrankungen wurde wiederholt als „neglected epidemic“ eingestuft [1, 13]. Ältere Menschen sind besonders häufig betroffen [7]. In Deutschland weisen zudem Menschen mit Pflegebedarf im Vergleich zu Gleichaltrigen ohne Pflegebedarf einen signifikant schlechteren Mund- und Zahnstatus auf [20].

Dennoch dominiert in Pflegeheimen in Deutschland eine beschwerdeorientierte zahnmedizinische Versorgung, mit der Präventionspotenziale nicht ausgeschöpft werden können [19, 23]. Zudem zeigen Mitarbeitende in Pflegeheimen ein eher geringes Interesse an der Mundgesundheit [11, 21]. Studien aus Deutschland wie auch anderen Ländern haben wiederholt darauf verwiesen, dass fehlende Zeitressourcen und mangelnde Kompetenzen im Pflegeheim einer Verbesserung der Mundgesundheit entgegenstehen [5, 6, 9, 24].

Mittlerweile wurden in Deutschland Maßnahmen zur Verbesserung der Mundgesundheit von älteren Menschen mit Pflegebedarf wie der interprofessionelle Expertenstandard „Förderung der Mundgesundheit in der Pflege“ [5, 24] sowie Kooperationsverträge zwischen Vertragszahnärzt*innen und Pflegeeinrichtungen nach § 119b Abs. 1 SGB V eingeleitet. Ihre Entfaltungsmöglichkeiten werden in der Versorgungspraxis entschieden. Wie sich hier die Chancen der Zusammenarbeit von Zahnärzt*innen und Pflegefachpersonen darstellen, ist Thema unserer Studie.

### Verbesserung der Mundgesundheit durch interprofessionelle Zusammenarbeit

Es gilt als unbestritten, dass die Mundgesundheit von Menschen in Pflegeheimen durch professionsübergreifende Zusammenarbeit gestärkt werden kann, denn „(…) it is neither an aged care problem nor a dental problem; it is a shared problem that calls for collaboration“ [17, S. 98]. Eine Reihe von Studien bestätigt, dass die interprofessionelle Zusammenarbeit von Zahnmedizin und Pflege mit einer höheren Lebensqualität bei den Bewohner*innen und der Reduktion von Krankenhausaufenthalten sowie Mortalitätsraten dieser assoziiert ist [3, 6, 10, 22]. Mehrere Untersuchungen zeigen, dass zahnärztliche Praxisteams und Mitarbeitende in Heimen eine hohe Bereitschaft haben, intensiver miteinander zu kooperieren [9, 10, 25].

International gibt es unterschiedliche Ansätze zur Stärkung der interprofessionellen Zusammenarbeit. Viele werden durch die Etablierung einer erweiterten Pflegerolle verankert: Pflegefachpersonen werden beispielsweise zu „oral health champions“ (Kanada) [15] oder „oral health coordinators“ (New Hampshire) [22] weitergebildet. Sie koordinieren die Durchführung der Mundhygiene in den Heimen wie auch die Besuche der Zahnärzt*innen [2, 15, 22]. Hierzu führen sie Mundassessments durch und entwickeln auf dieser Basis individuelle Mundpflegepläne [2, 15, 22]. Ein systematisches Review, welches die Wirksamkeit unterschiedlicher (inter)professioneller Interventionen vergleichend untersucht hat, zeigt, dass insbesondere die Zusammenarbeit einer auf Mundgesundheit spezialisierten Pflegefachperson mit einem interdisziplinären Team zu einer verbesserten Mundgesundheit der Bewohner*innen führt [3].

### Ziel und Fragstellung

Angesichts der Potenziale interprofessioneller Zusammenarbeit und erweiterter Pflegerollen für die Verbesserung der Mundgesundheit von Heimbewohner*innen, zielte unsere Studie darauf, Sichtweisen von Zahnärzt*innen und Pflegefachpersonen in Deutschland auf ihre Zusammenarbeit im Pflegeheim zu analysieren. Dabei standen als Fragen im Fokus, a) welche Möglichkeiten und Herausforderungen Pflegefachpersonen und Zahnärzt*innen in der Zusammenarbeit im Pflegeheim sehen, und b) inwiefern sie sich eine intensivere Zusammenarbeit in der Zukunft vorstellen können.

## Methodik

Wir haben Experteninterviews [18] mit Zahnärzt*innen und Pflegefachpersonen durchgeführt, um ihre Sichtweisen auf die aktuelle und zukünftige Zusammenarbeit von Zahnmedizin und Pflege im Pflegeheim zu analysieren.

### Leitfaden und Sampling

Der Leitfaden fokussierte nach zwei offenen Einstiegsfragen in drei thematischen Blöcken das Betriebs- und Kontextwissen der Befragten [18]: a) Status quo der interprofessionellen Zusammenarbeit; b) Möglichkeiten und Herausforderungen einer intensiveren Zusammenarbeit und Aufgabenübertragung von Zahnärzt*innen auf Pflegefachpersonen; c) Ideale, Wünsche und Blick in die Zukunft. Die Interviewfragen wurden in Anlehnung an das episodische Interview entwickelt [8, S. 287 f.]. Hierzu wurden zielgerichtete Fragen zur Erhebung semantischen Wissens mit der Aufforderung verbunden, spezifische Situationen zu schildern, um narrativ-episodisches Wissen zu erheben. Abgesehen von allgemeinen Fragen zum Thema Aufgabenübertragung von Zahnärzt*innen auf Pflegefachpersonen im Rahmen künftiger Zusammenarbeit wurden die Interviewten nicht nach spezifischen Kooperationsformen oder Pflegerollen gefragt (Zusatzmaterial online: Leitfaden).

Die Gewinnung von Studienteilnehmer*innen beschränkte sich auf die Region Westfalen-Lippe (ca. 8,3 Mio. Einwohner*innen). Ziel der Samplingstrategie war es, Interviewpartner*innen zu gewinnen, die sich nach Auskunft Dritter durch Interesse und Engagement in der Zahn- und Mundgesundheit von Heimbewohner*innen auszeichnen. Die dahinterliegende Erwartung war, dass diese über eine besondere Expertise im Thema verfügen und daher geeignet sind, Visionen für eine zukünftige interprofessionelle Zusammenarbeit beider Berufsgruppen zu entwickeln. Im ersten Schritt erfolgte die Ansprache von Zahnärzt*innen auf Basis von Empfehlungen der Kassenzahnärztlichen Vereinigung Westfalen-Lippe (KZVWL). Die Pflegefachpersonen wurden auf Basis von Empfehlungen der interviewten Zahnärzt*innen sowie einer bekannten Pflegefachperson angesprochen. Die potenziellen Interviewpartner*innen wurden per E‑Mail, Telefon oder persönlich kontaktiert und mit einer Teilnahmeinformation über die Studie aufgeklärt. Eingeschlossen wurden Zahnärzt*innen und Pflegefachpersonen, die ihren Beruf a) aktiv, b) seit mindestens 2 Jahren ausüben und c) im regelmäßigen Kontakt zur jeweils anderen Berufsgruppe stehen sowie d) im Vorgespräch Interesse an der Weiterentwicklung der Zusammenarbeit von Zahnmedizin und Pflege in Pflegeheimen geäußert haben.

Insgesamt wurden 4 Pflegefachpersonen, darunter 2 Pflegedienstleitungen, aus stationären Pflegeeinrichtungen sowie 4 Zahnärzt*innen interviewt (Tab. 1).

**Table 1:** 

	Pflegefachpersonen	Pflegedienstleitungen	Zahnärzt*innen	Gesamt
*Weiblich*	2	2	3	7
*Männlich*	0	0	1	1
*Gesamt*	**2**	**2**	**4**	**8**

### Interviewdurchführung und -auswertung

Die Interviews wurden von der Erstautorin^1^ im Juni und Juli 2021 aufgrund der COVID-19-Pandemie telefonisch oder via Zoom durchgeführt und dauerten 24 bis 56 min. Keine*r der Befragten berichtete während des Interviews aus einem Modellprojekt der zahnärztlichen Versorgung oder interprofessionellen Zusammenarbeit im Pflegeheim. Die Interviewpartner*innen beziehen sich ausschließlich auf ihre Erfahrungen in der Regelversorgung.

Die Audioaufnahmen wurden vollständig transkribiert und die pseudonymisierten Transkripte mittels MAXQDA (VERBI) von der Erstautorin mittels thematischen Kodierens ausgewertet [8, S. 476ff.]. Hierzu wurden zunächst jedes Interview offen kodiert [26] sowie fallbezogen Kategorien gebildet und jeweils eine fallbezogene Vignette erstellt [8]. Zur fallvergleichenden Analyse wurden die zuvor gebildeten Kategorien der einzelnen Fälle verglichen und in einer gemeinsamen fallübergreifenden thematischen Struktur, d. h. ein gemeinsames Kategoriensystem, entlang folgender Oberkategorien zusammengeführt: (a) Zahn- und Mundgesundheitsversorgung im Setting Pflegeheim; (b) (fehlendes) Wissen und Problembewusstsein über Mundhygiene bei der Pflege; (c) Bewohner*innen mit besonderem Unterstützungsbedarf; (d) Möglichkeiten und Chancen der intensiveren Zusammenarbeit.

Für die Fragestellung dieses Artikels wurde im Autorenteam für die Kategorie „Möglichkeiten und Chancen der intensiveren Zusammenarbeit“ unter Anwendung des Strauss’schen Kodierparadigmas [26] eine detaillierte Interpretation vorgenommen. Es wurden 2 Subkategorien herausgearbeitet, die im Folgenden dargelegt werden: (d1) zukünftig erweiterte Rolle für Pflegefachpersonen; (d2) stärkere Verankerung (zahn)medizinischer Versorgung in interprofessioneller Zusammenarbeit.

## Ergebnisse

### Zukünftig erweiterte Rolle für Pflegefachpersonen

Für die Verbesserung der zahnärztlichen Behandlung im Pflegeheim sehen die befragten Zahnärzt*innen Pflegefachpersonen als entscheidende Ansprechpartner*innen an:* „(…) denn ohne die Pflege[fach]kräfte funktioniert das einfach nicht, weil die den Patienten jeden Tag sehen“* (Z^2^). Die Pflegefachpersonen nehmen bereits heute in der Zusammenarbeit oft eine Art Stellvertreterfunktion für die Bewohner*innen ein, wenn diese ihre Anliegen selbst nicht mehr kommunizieren können.

Aus Sicht der Befragten beider Berufsgruppen ist es jedoch notwendig, dass Pflegefachpersonen „*auch extra geschult werden, dass (…) einer weiß, worum es geht und es weitersagt und es auch kontrolliert“* (P). Sie sollten dann als Multiplikator*innen dazu beitragen, dass das oft vernachlässigte Thema der Mundhygiene stärker in den Fokus der Grundpflege rückt, wo *„manchmal die Entscheidung [fällt] ,rasiere ich heute den Patienten oder putze ich ihm die Zähne’ und dann wird sich manchmal fürs Rasieren entschieden“* (Z).

Denn neben dem bekannten Zeitmangel in der Pflege ist aus Sicht aller Befragten ein geringer Wissensstand in Pflegeheimen zur Mundgesundheit und -hygiene festzustellen, der durch ein gering ausgeprägtes Problembewusstsein von Betroffenen, Angehörigen, aber auch der Pflegefachpersonen selbst noch verstärkt werde. Dies erschwere die Zusammenarbeit von Zahnärzt*innen und Pflegefachpersonen; das Thema Mundgesundheit werde in der Pflegeausbildung *„nicht vermittelt“* (P).

Als Möglichkeit, den derzeitigen Herausforderungen entgegenzuwirken, können sich die Befragten eine erweiterte Rolle für Pflegefachpersonen vorstellen – *„warum sollte es keinen Mund-Manager geben?“* (Z), bzw. eine* „Mundhygienespezialistin“* (P). Sie ziehen hier den Vergleich mit der Dekubitusprophylaxe der Wundmanager*innen im Pflegeheim. Ein*e solche*r Mundhygieneexpert*in könne eine individuell verbesserte Mundhygienesituation der Heimbewohner*innen verantworten und dafür sorgen, dass der Behandlungsbedarf sowie Notfälle reduziert werden. Die Befragten versprechen sich davon im eng getakteten Arbeitsalltag eine* „Entlastung für beide Seiten“* (Z).

### Stärkere Verankerung (zahn‑)medizinischer Versorgung in interprofessioneller Zusammenarbeit

Verbunden mit den Ideen einer verstärkten Verantwortungsübernahme von Pflegefachpersonen zur Förderung der Mundgesundheit beschreiben die Befragten auch ein Heranrücken von Pflegeeinrichtungen an medizinische Versorgungsaufgaben, was aus ihrer Sicht eine interprofessionelle Zusammenarbeit erforderlich macht. Diese sollte aus Sicht der Befragten Zugangsbarrieren für Bewohner*innen mit besonderem Unterstützungsbedarf abbauen. Insbesondere Menschen mit Demenz sollten für medizinische Behandlungen *„möglichst wenig die Einrichtung verlassen“* (P), um unnötige Belastungen zu vermeiden. Daher ist es für die Befragten zentral, die zahnmedizinische Behandlung *in* der Pflegeeinrichtung strukturell zu verankern. Die Zahnärzt*innen haben die zusätzliche Schwierigkeit, dass Menschen mit Demenz Schmerzen in der Mundhöhle oft nicht äußern und abwehrendes Verhalten zeigen. Die Befragten sind sich darin einig, dass die zahnärztliche Behandlung im Pflegeheim daher individuell durch Pflegefachpersonen begleitet werden sollte, die ihre Expertise im Umgang mit den Bewohner*innen einbringen können; eine gemeinsame Konsultation würde laut den Befragten den fachlichen Austausch zwischen beiden Professionen ermöglichen.

Speziell die Zahnärzt*innen betonen, dass Pflegefachpersonen bei Menschen mit Demenz die Mundhöhle verstärkt beobachten sollten, um frühzeitig Probleme erkennen zu können. Voraussetzung dafür sind aus ihrer Sicht Rahmenbedingungen, die eine entsprechende Qualifizierung und Vergütung dieser spezialisierten Fachkräfte regeln. Unter diesen Voraussetzungen können sich neue Kooperationsmuster zwischen Pflegefachpersonen und Zahnärzt*innen entwickeln. Dabei braucht es aus Sicht der Zahnärzt*innen Klarheit darüber, wie sich die Zusammenarbeit gestalten lässt.

Die Zahnärzt*innen schildern diesbezüglich, dass regelmäßigere Treffen mit Pflegefachpersonen stattfinden müssen, um gegenseitiges Feedback zu üben und Abläufe der Zusammenarbeit kontinuierlich zu evaluieren und optimieren. Die Pflegefachpersonen können sich vorstellen, dass regelmäßige Schulungsangebote von Zahnärzt*innen hilfreich wären, um Kompetenzen zu erweitern und das Bewusstsein zu stärken. Eine interprofessionelle Zusammenarbeit könne darüber hinaus durch den Austausch auch mit weiteren Berufsgruppen, z. B. Hausärzt*innen oder Neurolog*innen, vorangebracht werden, um grundsätzliche Fragen der Medikation bei zahnärztlichen Eingriffen abzustimmen.

Für eine Verankerung der Mund- und Zahngesundheit in der Pflegeeinrichtung kommt es aus Sicht der Befragten aber auch darauf an, dass weniger eine Vielzahl an Verantwortlichen und vielmehr ein*e einzige*r Ansprechpartner*in die Zusammenarbeit stärken kann: *„so ein Manager, dass* **eine** *Person sich um (…) die Zähne [kümmert] oder die anderen um die Wunden, und nicht so, dass eine auf allen Hochzeiten tanzt, die am Ende* **nichts richtig** **und immer** *gemacht hat“* (P).

*„Also diese Zusammenarbeit muss wirklich Hand in Hand gehen, wie man sich ein Netzwerk vorstellt, und nicht nur wir mit dem Pflegepersonal oder mit der Pflegedienstleitung, sondern genauso mit den Hausärzten und den anderen Akteuren in dem Haus, (…) und das Zentrum ist immer der Bewohner. […] Und das hilft natürlich immer, wenn man (…) nicht fünf Ansprechpartner hat, sondern einen, und der aber wiederum seine Leute dann informiert und einstielt in die Richtung, wo es hingehen soll.“* (Z)

## Diskussion

Ziel dieser Studie war es, Sichtweisen von Zahnärzt*innen und Pflegefachpersonen auf ihre aktuelle und künftige Zusammenarbeit in Pflegeheimen zu untersuchen. Mit Blick auf die aktuelle Zusammenarbeit wird deutlich, dass die Befragten, wenngleich sie diese schätzen, zeitliche, personelle und strukturelle Herausforderungen im Pflegeheim als limitierend für eine aus ihrer Sicht notwendige Verbesserung der interprofessionellen Zusammenarbeit zur Förderung der Mundgesundheit von Heimbewohner*innen ansehen.

### Eine erweiterte Pflegerolle als Anker zur Verbesserung der Mund- und Zahngesundheit im Pflegeheim

Ausgehend von dem Befund, dass in Deutschland im aktuellen Pflegeheimalltag die Mund- und Zahngesundheit bisher nur schwer verankert werden konnte, entwickeln die Befragten die Idee bzw. den Wunsch nach einer Spezialisierung einzelner Pflegefachpersonen als „Mundmanager*innen“. Das Aufgaben- und Rollenprofil, das die Interviewpartner*innen hier zeichnen, ähnelt den Beschreibungen erweiterter Pflegerollen in internationalen Studien [2, 3, 10, 22]. Im Einklang mit den internationalen Studienergebnissen erwarten die Interviewpartner*innen unserer Studie, dass eine stärkere Verantwortungsübernahme von Pflegefachpersonen in erweiterter Rolle zur Verbesserung der Mundgesundheit beiträgt und auch in Deutschland die Qualität der Zusammenarbeit von Pflege und Zahnmedizin steigern könnte. Zugleich betonen sie die Notwendigkeit einer klaren Regelung und Definition der Aufgaben- und Verantwortungsbereiche einer solchen erweiterten Pflegerolle. Das neue Pflegeberufegesetz in Deutschland ermöglicht eine entsprechende Definition vorbehaltener Tätigkeiten (§ 4 PflBG). Dabei ist eine angemessene Qualifikation von Pflegefachpersonen in erweiterter Rolle zu gewährleisten. In den referierten internationalen Studien sind Pflegefachpersonen in der Regel grundständig hochschulisch qualifiziert. Zukünftige Forschung zu und Entwicklung von erweiterten Rollen von Pflegefachpersonen im Bereich Mundgesundheit sollte hier ansetzen und überprüfen, inwiefern erweiterte Pflegerollen im Bereich Mund- und Zahngesundheit mit einer Mindestqualifikation auf Bachelor- oder sogar Masterniveau hinterlegt werden sollten.

Eine Vielzahl an Studien belegt darüber hinaus, dass spezielle Fortbildungen zur Mundgesundheit das Wissen von Pflegefachpersonen verbessern und eine optimierte Mundhygiene bei den Heimbewohner*innen ermöglichen [4, 9, 11, 16, 27]. Die Ergebnisse unserer Studie verdeutlichen hier, dass neben dem Wissenszuwachs Einzelner vor allem der Transfer in das und die Zusammenarbeit mit dem Pflegeteam als grundlegend für die Förderung der Mundgesundheit im Pflegeheim angesehen werden. Wie die Befragten unserer Studie betonen, ist grundlegendes Wissen auch in der Breite nötig, die Kompetenzerweiterung innerhalb der Ausbildung wird als entscheidend eingestuft [4]. Andernfalls dürfte die Funktion des*der Mundmanager*in in Pflegeheimen wenig wirkungsmächtig sein [17].

### Integrierende Modelle der medizinischen Versorgung in Pflegeheimen

Neben der Verankerung im Pflegeteam ist aus Sicht der Befragten die interprofessionelle Zusammenarbeit mit Zahnärzt*innen wesentlich für die Förderung der Mundgesundheit von Heimbewohner*innen. Auch dies steht im Einklang mit der aktuellen Studienlage [14], wobei entwickelte Konzepte in Deutschland noch in den Kinderschuhen stecken.

Ähnlich zu einer aktuellen Studie in Deutschland, die die Zusammenarbeit von Haus- und Fachärzt*innen mit dem Pflegeheimpersonal untersucht [12], betonen auch die Interviewpartner*innen dieser Studie Vorteile optimierter Kommunikationswege mit festen Ansprechpersonen sowie kontinuierlich stattfindenden Treffen und Fallbesprechungen. Die Interviewpartner*innen erachten es darüber hinaus als sinnvoll, weitere Berufsgruppen, wie z. B. Hausärzt*innen, einzubinden, um die Heimbewohner*innen professionsübergreifend zu versorgen.

Die Ergebnisse unserer Studie zeigen, dass Fragen der Verbesserung der zahnmedizinischen Versorgung von Bewohner*innen in Pflegeheimen im breiteren Kontext einer Neuorientierung und verstärkten Integration von medizinischer Versorgung in Pflegeheimen betrachtet und analysiert werden sollten. Anstelle punktueller Verbesserungen einzelner medizinischer Interventionen erscheint es erforderlich, integrierende Modelle der medizinischen Versorgung in Pflegeheimen zu entwickeln. Der Pflege kommt dabei ein entscheidender Gestaltungsauftrag zu.

### Limitationen

Es wurden Teilnehmende in die Studie eingeschlossen, die von Dritten aufgrund ihres erkennbaren Engagements für eine verbesserte Mundgesundheit von Pflegeheimbewohner*innen empfohlen wurden. Alle Studienteilnehmer*innen zeigten sich zudem im Interview generell offen und positiv gegenüber einer Neuordnung von Aufgaben und intensiveren Zusammenarbeit zwischen Zahnärzt*innen und Pflegefachpersonen. Wir sehen die gewählte Samplingstrategie als sinnvoll an, um Potenziale für die Weiterentwicklung und Innovationsfähigkeit der interprofessionellen Zusammenarbeit von Zahnmediziner*innen und Pflegefachpersonen zu eruieren. Allerdings ist zu betonen, dass über eine andere Samplingstrategie gewonnenes Datenmaterial vermutlich mehr Herausforderungen und weniger Ideen für neue Formen der Zusammenarbeit zwischen Zahnärzt*innen und Pflegefachpersonen aufgezeigt hätte.

## Fazit für die Praxis

- Die Studienergebnisse verweisen auf das hohe Potenzial einer intensiveren Zusammenarbeit von Zahnärzt*innen und Pflegefachpersonen für die Verbesserung der Mundgesundheit von Bewohner*innen von Pflegeheimen.
- Qualifizierungsmaßnahmen in der Mundhygiene für Pflegefachpersonen sollten durch Definition neuer Pflegerollen und die Einführung regelhafter Austauschformate zur Klärung der Aufgaben und Rollen zwischen Zahnärzt*innen und Pflegefachpersonen flankiert werden.

### Supplementary Information





## Footnotes

1 Diese Studie basiert auf der Masterarbeit der Erstautorin im Masterstudiengang M.Sc. Public Health der Universität Bielefeld, die durch die Co-Autor*innen betreut wurde. Studiendesign und -konzept sowie der Leitfaden wurden in enger Abstimmung mit der Letztautorin (Versorgungsforschung/Pflegewissenschaft) entwickelt und zudem mehrfach mit der Co-Autorin (Rehabilitationswissenschaft) diskutiert, die beide über umfangreiche Erfahrungen in der qualitativen Sozialforschung verfügen. Für den vorliegenden Artikel wurden eine Fokussierung auf eine Teilfragestellung sowie vertiefende Analysen und Interpretationen des ursprünglich im Rahmen der Masterarbeit analysierten Datenmaterials im gesamten Autorenteam vorgenommen.

2 Z = Aussagen der Zahnärzt*innen; P = Aussagen der Pflegefachpersonen und Pflegedienstleitungen.
