# Supplementary material for: Oral health in nursing homes as an interprofessional task: Results of qualitative interviews with nurses and dentists on their current and future collaboration
Source: Z Gerontol Geriatr. 2022 Nov 7;56(8):667–72. [Article in German] doi: 10.1007/s00391-022-02132-5 (PMC9640861; doi:10.1007/s00391-022-02132-5)
Supplement: Supplementary file 1 [file 391_2022_2132_MOESM1_ESM.pdf]

Leitfaden für Experteninterviews mit Zahnärzt*innen und Pflegefachpersonen

Einstiegsfragen

Können Sie sich kurz vorstellen und für den Einstieg etwas über Ihren beruflichen Werdegang und Ihre Aufgaben erzählen?

Können Sie mir einen typischen Arbeitstag schildern, damit ich mir Ihre Tätigkeiten besser vorstellen kann? Wie verlief zum Beispiel ihr Arbeitstag gestern (oder an einem anderen Tag, wenn es gestern untypisch verlaufen ist)

Status Quo der interprofessionellen Zusammenarbeit

Inwieweit stehen Sie in Ihrem Berufsalltag in Kontakt mit Pflegefachpersonen aus stationären Pflegeeinrichtungen/ Zahnärzt*innen? Können Sie mir typische Anlässe und Situationen schildern, wo sie in Kontakt stehen?

- Gibt es noch weitere Anlässe/Situationen? Können Sie mir auch diese näher beschreiben?

Wie kann ich mir dann die genaue Zusammenarbeit zwischen Ihnen und den Pflegefachpersonen/ Zahnärzt*innen genauer vorstellen?

- Könnten Sie mir das anhand eines Beispiels schildern, wo Sie in einem (regelmäßigen) Austauschprozess mit Pflegefachperson/ Zahnärzt*innen sind?

Wenn Sie einmal an bestimmte Bewohner*innen denken – gibt es welche, bei denen eine verstärkte Zusammenarbeit zwischen Ihnen und den Pflegefachpersonen/ Zahnärzt*innen stattfindet?

- Könnten Sie mir dazu unterstützend einen Fall geben?
- wenn ja* warum genau diese Bewohner*innen? Fallen Ihnen weitere Bewohner*innen ein?

Was würden Sie sagen, klappt aktuell gut in der Zusammenarbeit mit den Pflegefachpersonen/ Zahnärzt*innen? Gibt es da Anhaltspunkte, die Sie mir an mehreren Beispielen erklären können?

Und umgekehrt, was würden Sie sagen, klappt weniger gut in der Zusammenarbeit? Erinnern Sie sich an bestimmte Situationen, die eher negativ waren?

Alles in Allem* Wie bewerten Sie insgesamt die Zusammenarbeit zwischen Ihnen und den Pflegefachpersonen/ Zahnärzt*innen?

Möglichkeiten und Herausforderungen einer intensiveren Zusammenarbeit und Aufgabenübertragung von Zahnärzt*innen auf Pflegefachpersonen

Wie könnte aus Ihrer Sicht die Zusammenarbeit zwischen Zahnärzt*innen und Pflegefachpersonen verbessert werden?

- Was könnten die Zahnärzt*innen und Pflegefachpersonen mehr/ anders leisten?

Gibt es allgemeine Bereiche in der Zahn- und Mundgesundheit und Mundhygiene bei den Bewohner*innen, an denen aus Ihrer Sicht Pflegefachpersonen sich künftig stärker beteiligten könnten? Könnten Sie mir hierzu Beispiele anführen?

Welche genauen Aufgaben könnten aus Ihrer Sicht Pflegefachpersonen übernehmen, die die Zahn-und Mundgesundheit der Bewohner*innen fördern?

- Könnten Sie dies an Beispielen verdeutlichen, die ihnen schon mal in den Sinn gekommen sind?
- Wie könnte dann die Zusammenarbeit zwischen Pflege und Zahnärzt*innen ausgestaltet werden?

Welche Vorteile und Chancen könnten sich aus Ihrer Sicht durch eine vermehrte Zusammenarbeit mit den Pflegefachpersonen/ Zahnärzt*innen und

Aufgabenübertragungen bei der Pflege für Ihre Arbeit als Pflegefachperson/ Zahnarzt*Zahnärztin ergeben?

- Können Sie mir das vielleicht anhand einer Erfahrung, die sie selbst gemacht haben, verdeutlichen?

Welche Vorteile und Chancen könnten sich aus Ihrer Sicht durch eine vermehrte Zusammenarbeit beider Professionen und die Aufgabenübertragungen der Pflege für die Bewohner*innen ergeben?

- Welche Bewohner*innen würden im Besonderen profitieren?

Welche Nachteile und Herausforderungen könnten sich aus Ihrer Sicht durch eine vermehrte Zusammenarbeit beider Professionen sowie die Aufgabenübertragung bei der Pflege für Ihre Arbeit ergeben?

Welche Nachteile und Herausforderungen könnten sich aus Ihrer Sicht durch eine vermehrte Zusammenarbeit mit den Pflegefachpersonen/ Zahnärzt*innen für die Bewohner*innen ergeben?

Welche Voraussetzungen müssen aus Ihrer Sicht für eine intensivere Zusammenarbeit beider Professionen und für eine Aufgabenübertragung bei der Pflege gegeben sein?

- Welche Rahmenbedingungen müssten dafür gegeben sein?
- Können Sie hier ein Beispiel nennen?

Ideale, Wünsche und Abschluss

Was wünschen Sie sich bezüglich der Zusammenarbeit von Zahnärzt*innen und Pflegefachpersonen für die Zukunft? Wie könnte Ihrer Ansicht nach eine Idealvorstellung der Zusammenarbeit aussehen?

Nun bin ich mit meinen Fragen am Ende angekommen. Gibt es aus Ihrer Sicht noch Aspekte/Themen, die ich noch nicht angesprochen haben, auf die Sie jedoch gerne noch eingehen würden?
